# Nationwide variations in the execution of minimally invasive right hemicolectomy and short-term outcomes: first phase of the RIGHT study

**DOI:** 10.1093/bjs/znae291

**Published:** 2024-11-18

**Authors:** Alexander A J Grüter, Willemijn A Jongsma, Nicola Leone, Hasti Barai, Boudewijn R Toorenvliet, Pieter J Tanis, Jurriaan B Tuynman, RIGHT Collaborators Group, S van Aalten, F Aarts, G S A Abis, B Akmaz, C S Andeweg, A H Baan, C I M Baeten, V P Bastiaenen, O Bastian, E H J Belgers, E J T Belt, B Berndsen, J Blauw, M Blussé van Oud-Alblas, F C den Boer, E J G Boerma, M D M Bolmers, R J I Bosker, J J van den Broek, S M M de Castro, I M Cherepanin, S H E M Clermonts, R J S Coelen, E C J Consten, A Demirkiran, E B Deerenberg, R Dijkstra, P van Duijvendijk, Y El-Massoudi, J A van Essen, D J Evers, H F J Fabry, S Fransen, P van Gerven, H Goei, J Gooszen, J Govaert, F A B Grimme, E J de Groof, B Grotenhuis, A den Hartog, T van Heek, J Heemskerk, B H M Heijnen, C Hoff, R Hompes, C D P van ‘t Hullenaar, G M de Jong, F H W Jonker, M R Ketting, J J S Kiewiet, J L M Konsten, S A Koopal, R T J Kortekaas, E Lagae, B Lamme, J W Leijtens, T Lettinga, H E Lont, T Lubbers, H A Marsman, J P Meekel, D J L de Mey, D E Moes, P A Neijenhuis, L C F de Nes, J Nonner, L E Nooijen, J M T Omloo, S E van Oostendorp, S J Oosterling, B Polle, A Pronk, R J Renger, M de Roos, J E Rütter, A P Schouten van der Velden, B P Smalbroek, A B Smits, E J Spillenaar Bilgen, E J A Steller, H B A C Stockmann, J H M B Stoot, J Straatman, H A Swank, Y K Sze, K Talsma, Q R J G Tummers, S C Veltkamp, A W H van de Ven, T Verhagen, P M Verheijen, M Vermaas, W J Vles, R J de Vos tot Nederveen Cappel, D K Wasowicz, M Westerterp, H L van Westreenen, K P Wevers, C D M Witjes, F T W E van Workum, R J Zijlstra, D D E Zimmerman

**Affiliations:** Department of Surgery, Amsterdam UMC location Vrije Universiteit Amsterdam, Amsterdam, The Netherlands; Treatment and Quality of Life, Cancer Center Amsterdam, Amsterdam, The Netherlands; Department of Surgery, Amsterdam UMC location Vrije Universiteit Amsterdam, Amsterdam, The Netherlands; Treatment and Quality of Life, Cancer Center Amsterdam, Amsterdam, The Netherlands; Department of Surgery, Amsterdam UMC location Vrije Universiteit Amsterdam, Amsterdam, The Netherlands; Department of Surgical Sciences, University of Turin, Turin, Italy; Department of Surgery, Amsterdam UMC location Vrije Universiteit Amsterdam, Amsterdam, The Netherlands; Department of Surgery, Ikazia Hospital, Rotterdam, The Netherlands; Department of Surgery, Amsterdam UMC location University of Amsterdam, Amsterdam, The Netherlands; Department of Surgery, Erasmus MC, Rotterdam, The Netherlands; Department of Surgery, Amsterdam UMC location Vrije Universiteit Amsterdam, Amsterdam, The Netherlands

Although minimally invasive right hemicolectomy (MIRH) has become the standard of care to treat patients with right-sided colon cancer, substantial variation in the execution and implementation of proven beneficial elements that impact clinical outcomes exists. Within the Dutch national RIGHT project, a Delphi consensus was conducted that established an evidence-based, standardized technique for MIRH, including: low intra-abdominal pressure (IAP), complete mesocolic excision (CME) with central dissection along the superior mesenteric vein (SMV) and central vascular ligation (CVL) of segmental vessels, an intracorporeal anastomosis, and specimen extraction through a Pfannenstiel incision (see *File S1* and *File S2*)^1,2^. The aim of the RIGHT project is to implement this standardized technique for MIRH nationwide in order to improve clinical outcomes. Within the first phase of the RIGHT study, the aim was to evaluate the nationwide variation of the elements of MIRH for right-sided colon cancer.

The RIGHT study is a multicentre national prospective cohort study, that started in October 2021 in the Netherlands, with 43 participating hospitals (43 of 71 = 60.6% of Dutch hospitals). Patients undergoing planned MIRH (both conventional laparoscopic and robot-assisted) for right-sided non-locally advanced stage 1–3 colon cancer were included. During the first phase (October 2021—August 2022), participating surgeons were instructed to perform the MIRH according to their routine practice. An essential aspect of this study is that surgeons were required to make a video recording of the entire procedure and to take a picture of both the front and back of the specimen. The quality of mesocolic excision was scored according to Benz, ranging from 0 to 3, where a lower score indicates a more extensive mesocolic excision, with Benz 0 corresponding to CME^3^.

A total of 414 patients were included in phase 1. See *Fig. S1* for the inclusion flowchart and *Table S1* for the baseline patient characteristics. *Table 1* provides an overview of all procedural variations within MIRH among the participating Dutch surgeons. The median IAP applied during surgery was 12 mmHg, ranging from 7 to 15 mmHg. Anastomoses were most often performed intracorporeally (80.9%), mostly in an isoperistaltic configuration (80.7%), and almost exclusively constructed using a stapler (96.3%). In 77.3% of cases, the specimen was extracted through a Pfannenstiel incision. The distribution of the Benz classification was as follows: 23% of cases were Benz 0, 30% of cases were Benz 1, 37% of cases were Benz 2, and 10% of cases were Benz 3. *Fig. S2* provides some examples of the different Benz categories. *Table S2* summarizes 90-day postoperative complications, which occurred in 25.9% of cases. Anastomotic leakage was noted in 2.7% of cases and the 90-day mortality rate was 0.7%. The median duration of hospital stay was 3 days and the readmission rate was 10.5%.

**Table 1:** Overview of variations within minimally invasive right hemicolectomy

Variation	Value
**Preoperative oral antibiotics, *n* of *n* (%)**	64 of 414 (15.5)
**Bowel preparation**	
None	370 (90.0)
Mechanical bowel preparation	36 (8.8)
Enema	4 (1.0)
Mechanical bowel preparation + enema	1 (0.2)
**Patient position**	
Supine	307 (75.6)
French	85 (20.9)
Lithotomy using stirrups	14 (3.4)
**Minimally invasive approach**	
Laparoscopic	382 (92.7)
Robot-assisted	30 (7.3)
**Number of trocars**	
3	20 (4.9)
4	334 (81.7)
5	50 (12.2)
>5	5 (1.2)
**Intra-abdominal pressure (mmHg), median (interquartile range; range)**	12 (12–12; 7–15)
**Approach for retroperitoneal dissection**	
Medial to lateral (through the ileal mesentery)	370 (90.5)
Lateral to medial	15 (3.7)
Caudal to cranial (subileal)	23 (5.6)
Cranial to caudal	1 (0.2)
**Indocyanine Green applied, *n* of *n* (%)**	68 of 410 (16.6)
**Anastomosis**	
Intracorporeal	331 (80.9)
Extracorporeal	78 (19.1)
Isoperistaltic	330 (80.7)
Antiperistaltic	79 (19.3)
Handsewn	15 (3.7)
Stapled	394 (96.3)
**Extraction site**	
Left lower quadrant	2 (0.5)
Transverse	16 (3.9)
Pfannenstiel	316 (77.3)
Right lower quadrant	7 (1.7)
Umbilical (midline)	49 (12.0)
Other	19 (4.6)
Specimen according to Benz classification*	
Benz 0	60 (23.0)
Benz 1	78 (30.0)
Benz 2	96 (36.9)
Benz 3	26 (10.0)

Values are *n* (%) unless otherwise indicated. *Benz classification: Benz 0 (true complete mesocolic excision specimen), the stalks of the ileocolic vessels and middle colic vessels are connected by tissue of the surgical trunk (lymphatic tissue package covering the superior mesenteric vein) and the mesocolic window has a complete medial frame of mesocolic tissue; Benz 1, the stalks of the ileocolic and middle colic vessels are present, but are not connected by tissue, and the frame of the mesocolic window is not complete with regard to its medial aspect; Benz 2, the stalks of the ileocolic vessels are present, with more than 50% of the anticipated length according to the geometric configuration of the specimen, but the middle colic vessels are not detectable, and the frame of the window has medial and cranial defects; and Benz 3, the ileocoloic vessels have an amputated appearance (less than 50% of the anticipated length according to the geometric configuration of the specimen) and the window is not detectable.

This first phase of the RIGHT study shows that, in the participating hospitals, a high percentage of MIRH operations are performed with the established evidence-based ‘new’ techniques (such as intracorporeal anastomosis and Pfannenstiel extraction) and with acceptable short-term morbidity. However, specimen evaluation demonstrated that a minority of the patients underwent a CME with central SMV dissection and CVL of segmental vessels, implying that this recent development and guideline recommendation is not yet broadly implemented in the Netherlands.

An intact mesocolon with central ligation of the vessels (optimal D2 dissection, thus CME) is believed to reduce the risk of recurrence and improve long-term survival^4,5^. Controversy exists in the literature regarding the definition of CME and whether a D3 dissection is an integral part of CME^6^. However, SMV dissection with CVL alone should result in a good-quality D2 dissection, resulting in a specimen that contains a surgical trunk medial to the supraduodenal window that connects the ileocolic and right colic pedicles. The recent RELARC trial did not show superiority regarding 3-year disease-free survival of extended D3 dissection *versus* D2 dissection^7^. However, both study groups underwent CVL with the aim of an intact mesocolic specimen. The fact that an optimal D2 dissection was achieved in only a minority of the patients in the present study highlights the importance of implementation programmes to help the surgical community to adapt to the recommendations of up-to-date guidelines.

An intracorporeal anastomosis was performed in a remarkably high proportion of patients. Several systematic reviews have highlighted the benefits of intracorporeal anastomosis *versus* extracorporeal anastomosis in MIRH, showing reduced short-term morbidity, decreased duration of hospital stay, and quicker recovery of bowel function^8^. Similarly, the use of a Pfannenstiel incision for specimen extraction in the present study is higher than generally reported. The previous literature consistently reports that Pfannenstiel extraction is advantageous for patients, with a lower incisional hernia rate compared with midline and other incisions^9^. The median IAP applied in this study was 12 mmHg. A recent RCT has demonstrated that maintaining a lower IAP at 8 mmHg, in comparison with 12 mmHg, resulted in decreased acute pain scores, a reduction in 30-day infectious complications, diminished surgical-site hypoxia and inflammatory markers, reduced postoperative cytokine production, and a higher-quality recovery^10^.

In the next phases of the RIGHT study, all key elements of the consensus-based standardized technique for MIRH will be included in training and proctoring, with continuous monitoring, including video recording, specimen pictures, CT imaging and clinical outcomes. This means that CME with SMV dissection and CVL will be performed using an 8 mmHg pneumoperitoneum, when possible, followed by an intracorporeal anastomosis with Pfannenstiel extraction.

In conclusion, during phase 1 of the RIGHT study, participating surgeons have already incorporated parts of the optimal technique for MIRH, such as intracorporeal anastomosis and Pfannenstiel extraction. However, there was still significant variability in certain steps, such as the extent of mesocolic excision and IAP. An optimal D2 dissection, and thus a CME, was achieved in only a minority of patients. This underscores the importance of the RIGHT project as a crucial initiative for the nationwide implementation of an optimized technique for MIRH, with the aim of improving both short- and long-term outcomes for patients with right-sided colon cancer.

## Collaborators

**RIGHT Collaborators Group**

S. van Aalten (Groene Hart Ziekenhuis, Gouda, the Netherlands); F. Aarts (VieCuri Medisch Centrum, Venlo, the Netherlands); G. S. A. Abis (Meander Medisch Centrum, Amersfoort, the Netherlands); B. Akmaz (Zuyderland Medisch Centrum, Heerlen, the Netherlands); C. S. Andeweg (Ziekenhuis St Jansdal, Harderwijk, the Netherlands); A. H. Baan (Amstelland Ziekenhuis, Amstelveen, the Netherlands); C. I. M. Baeten (Groene Hart Ziekenhuis, Gouda, the Netherlands); V. P. Bastiaenen (Flevoziekenhuis, Almere, the Netherlands); O. Bastian (Beatrixziekenhuis, Gorinchem, the Netherlands); E. H. J. Belgers (Zuyderland Medisch Centrum, Heerlen, the Netherlands); E. J. T. Belt (Albert Schweitzer Ziekenhuis, Dordrecht, the Netherlands); B. Berndsen (Spaarne Gasthuis, Haarlem, the Netherlands); J. Blauw (Alrijne Ziekenhuis, Leiderdorp, the Netherlands); M. Blussé van Oud-Alblas (Maasziekenhuis Pantein, Beugen, the Netherlands); F. C. den Boer (Zaans Medisch Centrum, Zaandam, the Netherlands); E. J. G. Boerma (Zuyderland Medisch Centrum, Heerlen, the Netherlands); M. D. M. Bolmers (Dijklander Ziekenhuis, Hoorn, the Netherlands); R. J. I. Bosker (Deventer Ziekenhuis, Deventer, the Netherlands); J. J. van den Broek (Dijklander Ziekenhuis, Hoorn, the Netherlands); S. M. M. de Castro (OLVG, Amsterdam, the Netherlands); I. M. Cherepanin (Bravis Ziekenhuis, Roosendaal, the Netherlands); S. H. E. M. Clermonts (Medisch Centrum Leeuwarden, Leeuwarden, the Netherlands); R. J. S. Coelen (Dijklander Ziekenhuis, Hoorn, the Netherlands); E. C. J. Consten (Meander Medisch Centrum, Amersfoort, the Netherlands); A. Demirkiran (Rode Kruis Ziekenhuis, Beverwijk, the Netherlands); E. B. Deerenberg (Franciscus Gasthuis, Rotterdam, the Netherlands); R. Dijkstra (St. Antonius Ziekenhuis, Nieuwegein, the Netherlands); P. van Duijvendijk (Gelre Ziekenhuizen, Apeldoorn, the Netherlands); Y. El-Massoudi (LangeLand Ziekenhuis, Zoetermeer, the Netherlands); J. A. van Essen (St. Jans Gasthuis, Weert, the Netherlands); D. J. Evers (Ziekenhuisgroep Twente, Hengelo, the Netherlands); H. F. J. Fabry (Bravis Ziekenhuis, Roosendaal, the Netherlands); S. Fransen (Laurentius Ziekenhuis, Roermond, the Netherlands); P. van Gerven (Flevoziekenhuis, Almere, the Netherlands); H. Goei (Zaans Medisch Centrum, Zaandam, the Netherlands); J. Gooszen (Flevoziekenhuis, Almere, the Netherlands); J. Govaert (Haaglanden Medisch Centrum, Den Haag, the Netherlands); F. A. B. Grimme (Nij Smellinghe, Drachten, the Netherlands); E. J. de Groof (Flevoziekenhuis, Almere, the Netherlands); B. Grotenhuis (Antoni van Leeuwenhoek Ziekenhuis, Amsterdam, the Netherlands); A. den Hartog (Antoni van Leeuwenhoek Ziekenhuis, Amsterdam, the Netherlands); T. van Heek (Ziekenhuis Gelderse Vallei, Ede, the Netherlands); J. Heemskerk (Laurentius Ziekenhuis, Roermond, the Netherlands); B. H. M. Heijnen (LangeLand Ziekenhuis, Zoetermeer, the Netherlands); C. Hoff (Medisch Centrum Leeuwarden, Leeuwarden, the Netherlands); R. Hompes (Amsterdam UMC location AMC, Amsterdam, the Netherlands); C. D. P. van ‘t Hullenaar (van Weel-Bethesda Ziekenhuis, Dirksland, the Netherlands); G. M. de Jong (Ziekenhuis Gelderse Vallei, Ede, the Netherlands); F. H. W. Jonker (Rode Kruis Ziekenhuis, Beverwijk, the Netherlands); M. R. Ketting (Van Weel-Bethesda Ziekenhuis, Dirksland, the Netherlands); J. J. S. Kiewiet (Diakonessenhuis, Utrecht, the Netherlands); J. L. M. Konsten (VieCuri Medisch Centrum, Venlo, the Netherlands); S. A. Koopal (Medisch Centrum Leeuwarden, Leeuwarden, the Netherlands); R. T. J. Kortekaas (Franciscus Gasthuis, Rotterdam, the Netherlands); E. Lagae (Stichting ZorgSaam Zeeuws Vlaanderen, Terneuzen, the Netherlands); B. Lamme (Albert Schweitzer Ziekenhuis, Dordrecht, the Netherlands); J. W. Leijtens (Laurentius Ziekenhuis, Roermond, the Netherlands); T. Lettinga (St. Jans Gasthuis, Weert, the Netherlands); H. E. Lont (Franciscus Gasthuis, Rotterdam, the Netherlands); T. Lubbers (Maastricht UMC+, Maastricht, the Netherlands); H. A. Marsman (OLVG, Amsterdam, the Netherlands); J. P. Meekel (Zaans Medisch Centrum, Zaandam, the Netherlands); D. J. L. de Mey (Stichting Zorgsaam Zeeuws Vlaanderen, Terneuzen, the Netherlands); D. E. Moes (Dijklander Ziekenhuis, Hoorn, the Netherlands); P. A. Neijenhuis (Alrijne Ziekenhuis, Leiderdorp, the Netherlands); L. C. F. de Nes (Maasziekenhuis Pantein, Beugen, the Netherlands); J. Nonner (Ikazia Ziekenhuis, Rotterdam, the Netherlands); L. E. Nooijen (Flevoziekenhuis, Almere, the Netherlands); J. M. T. Omloo (Gelre Ziekenhuizen, Apeldoorn, the Netherlands); S. E. van Oostendorp (Rode Kruis Ziekenhuis, Beverwijk, the Netherlands); S. J. Oosterling (Spaarne Gasthuis, Haarlem, the Netherlands); B. Polle (Canisius Wilhelmina Ziekenhuis, Nijmegen, the Netherlands); A. Pronk (Diakonessenhuis, Utrecht, the Netherlands); R. J. Renger (Beatrixziekenhuis, Gorinchem, the Netherlands); M. de Roos (Rijnstate Ziekenhuis, Arnhem, the Netherlands); J. E. Rütter (Rijnstate Ziekenhuis, Arnhem, the Netherlands); A. P. Schouten van der Velden (Ziekenhuis St Jansdal, Harderwijk, the Netherlands); B. P. Smalbroek (St. Antonius Ziekenhuis, Nieuwegein, the Netherlands); A. B. Smits (St. Antonius Ziekenhuis, Nieuwegein, the Netherlands); E. J. Spillenaar Bilgen (Rijnstate Ziekenhuis, Arnhem, the Netherlands); E. J. A. Steller (Isala Ziekenhuizen, Zwolle, the Netherlands); H. B. A. C. Stockmann (Spaarne Gasthuis, Haarlem, the Netherlands); J. H. M. B. Stoot (Zuyderland Medisch Centrum, Heerlen, the Netherlands); J. Straatman (Rode Kruis Ziekenhuis, Beverwijk, the Netherlands); H. A. Swank (OLVG, Amsterdam, the Netherlands); Y. K. Sze (Admiraal de Ruyter Ziekenhuis, Vlissingen, the Netherlands); K. Talsma (Deventer Ziekenhuis, Deventer, the Netherlands); Q. R. J. G. Tummers (Haaglanden Medisch Centrum, Den Haag, the Netherlands); S. C. Veltkamp (Amstelland Ziekenhuis, Amstelveen, the Netherlands); A. W. H. van de Ven (Flevoziekenhuis, Almere, the Netherlands); T. Verhagen (Ziekenhuisgroep Twente, Hengelo, the Netherlands); P. M. Verheijen (Meander Medisch Centrum, Amersfoort, the Netherlands); M. Vermaas (IJsseland Ziekenhuis, Rotterdam, the Netherlands); W. J. Vles (Ikazia Ziekenhuis, Rotterdam, the Netherlands); R. J. de Vos tot Nederveen Cappel (Admiraal de Ruyter Ziekenhuis, Vlissingen, the Netherlands); D. K. Wasowicz (Elisabeth-TweeSteden Ziekenhuis, Tilburg, the Netherlands); M. Westerterp (Haaglanden Medisch Centrum, Den Haag, the Netherlands); H. L. van Westreenen (Isala Ziekenhuizen, Zwolle, the Netherlands); K. P. Wevers (Maastricht UMC+, Maastrich, the Netherlands); C. D. M. Witjes (IJsselland Ziekenhuis, Rotterdam, the Netherlands); F. T. W. E. van Workum (Canisius Wilhelmina Ziekenhuis, Nijmegen, the Netherlands); R. J. Zijlstra (Nij Smellinghe, Drachten, the Netherlands); D. D. E. Zimmerman (Elisabeth-TweeSteden Ziekenhuis, Tilburg, the Netherlands).

## Supplementary Material

znae291_Supplementary_Data

## Data Availability

The study data are not openly available. The authors are willing to share the data, upon reasonable request.
