# Supplementary material for: Nationwide variations in the execution of minimally invasive right hemicolectomy and short-term outcomes: first phase of the RIGHT study
Source: Br J Surg. 2024 Nov 18;111(11):znae291. doi: 10.1093/bjs/znae291 (PMC11572717; doi:10.1093/bjs/znae291)
Supplement: znae291_Supplementary_Data [file znae291_supplementary_data.zip › Supplementary_File_1._Original_study_protocol_entire_Right_study.docx]

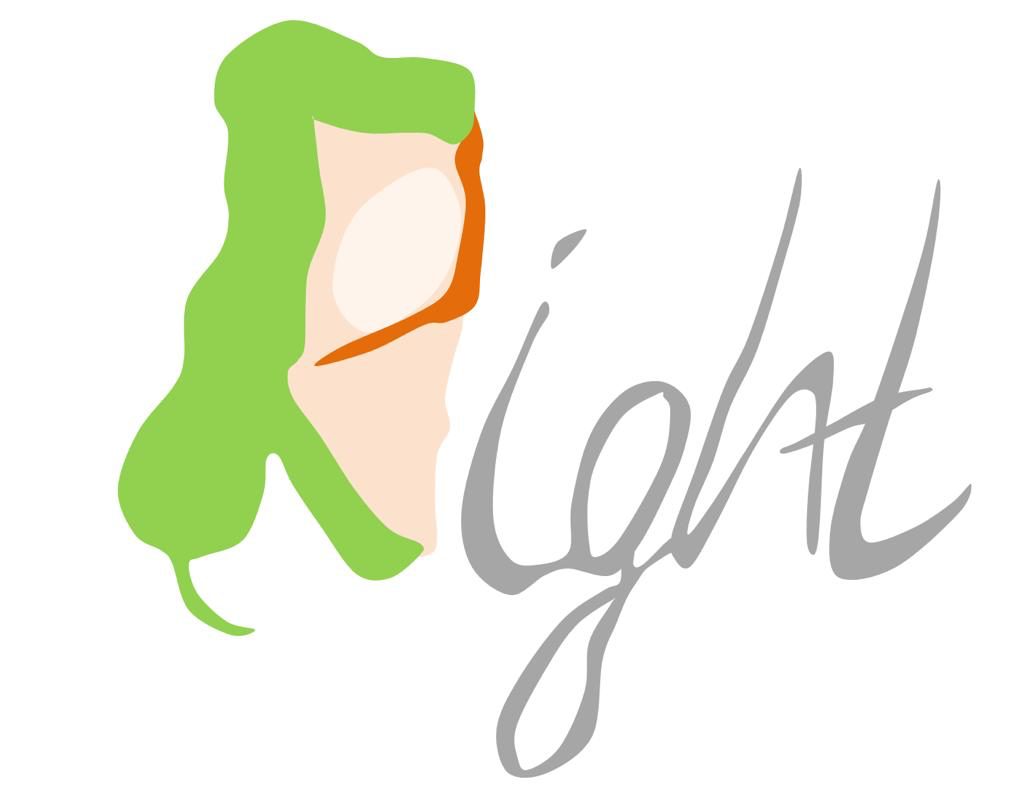


STUDY

**Implementation of optimized and standardized surgical technique for right-sided colon cancer: a prospective interventional sequential cohort study with a transition period**

**Principal Investigators**

Dr. J.B. Tuynman, colorectal surgeon

Dr. B.R. Toorenvliet, colorectal surgeon

Prof. dr. P.J. Tanis, colorectal surgeon

Steering committee: Dr. S. van Aalten, Dr. E.J.Th. Belt, Dr. F. den Boer, Prof. dr. H.J. Bonjer, Prof. dr. E.C.J. Consten, Dr. P. van Duijvendijk, Dr. W.M.U. van Grevenstein, Dr. C. Hoff, Prof. dr. G.J. Kleinrensink, Prof. dr. J. Lange, Drs. J. Leijtens, Drs. J. Nonner, Dr. C. Sietses, Dr. G. D. Slooter, Drs. A.W.H. van de Ven, Dr. M. Vermaas, Dr. H.L. van Westreenen, Dr D.D.E. Zimmerman.

International collaborators for Delphi process, training and proctoring:

- D. Miskovic, St Marks hospital London, UK

- S. Benz, Klinikverbund SuedWest, Böblingen, Germany

- F. Aigner, Krankenhaus der Barmherzigen Bruder, Graz, Austria

- C.A. Bertelsen, Hillerød University Hospital, Copenhagen, Denmark

Collaborators (coauthors); representatives from all participating centers.

Study coordinators:

- Drs. A.A.J. Grüter, PhD student
- Dr. U.K. Coblijn, surgical resident

**PROTOCOL TITLE:** Implementation of optimized and standardized surgical technique for right-sided colon cancer: a prospective interventional sequential cohort study with a transition period.

| **Protocol ID** | VUmc_2021-5600 |
| --- | --- |
| **Short title** | Right Study |
| **Version** | 2.0 |
| **Date** | October 2021 |
| **Principal investigators** | **Dr. J.B. Tuynman, project leader**  *Address*: Amsterdam UMC, location VUmc, Department of Surgery, De Boelelaan 1117, 1118, 1081 HV, Amsterdam  *Email*: [j.tuynman@amsterdamumc.nl](mailto:j.tuynman@amsterdamumc.nl)  *Telephone*: +31 630155307  **Dr. B.R. Toorenvliet**  *Address*: Ikazia Hospital Rotterdam, Department of Surgery, Montessoriweg 1, 3083 AN, Rotterdam  *Email*: [br.toorenvliet@ikazia.nl](mailto:br.toorenvliet@ikazia.nl)  Telephone: +31 650251180  **Prof. dr. P.J. Tanis**  *Address*: Amsterdam UMC, location VUmc, Department of Surgery, De Boelelaan 1117, 1118, 1081 HV, Amsterdam  *Email*: [p.tanis@erasmusmc.nl](mailto:p.j.tanis@amsterdamumc.nl)  *Telephone*: +31 629068275 |
| **Local principal investigators**   1. Dr. R.J. de Vos 2. Dr. E.J.T. Belt 3. Drs. P.A. Neijenhuis 4. Dr. S.C. Veltkamp 5. Dr. R. Hompes & dr. W. Laméris 6. Dr. J.B. Tuynman & prof. dr. P.J. Tanis 7. Dr. B. Grotenhuis 8. Drs. T. Brandsma 9. R.J. Renger & F. Logeman 10. Drs. H.F.J. Fabry 11. Dr. F.T.W.E. van Workum 12. Dr. R.J.I. Bosker 13. Dr. A. Pronk 14. Dr. D.J.A. Sonneveld 15. Dr. D.D.E. Zimmerman 16. Dr. A.W.H. van de Ven 17. Drs. R.Th.J. Kortekaas 18. Dr. P. van Duijvendijk 19. Dr. S. van Aalten 20. Dr. M. Westerterp 21. Dr. C. Sietses 22. Dr. T. Verhagen 23. Dr. A.P. Schouten van der Velden 24. Dr. M. Vermaas 25. Drs. J. Nonner 26. Dr. H.L. van Westreenen 27. Drs. Y. El Massoudi 28. Dr. J.W.A. Leijtens 29. Dr. K.C.M.J. Peeters 30. Dr. T. Lubbers 31. Dr. L. de Nes 32. Prof. dr. E.C.J. Consten 33. Dr. C. Hoff 34. Dr. P. Steenvoorde 35. Dr. I.T.A. Pereboom 36. Dr. R.N. van Veen 37. Dr. B.P.L. Witteman 38. Dr. H.A. Cense 39. Drs. T. Lettinga 40. Dr. S.J. Oosterling 41. Drs. D. de Mey 42. Dr. R.L. van der Hul 43. Dr. J.L.M. Konsten 44. Dr. F.C. den Boer 45. Dr. E. Boerma | 1. Admiraal de Ruyter Hospital, Vlissingen 2. Albert Schweitzer Hospital, Dordrecht 3. Alrijne Hospital, Leiderdorp, Leiden, Alphen aan den Rijn 4. Amstelland Hospital, Amstelveen 5. Amsterdam UMC, location AMC, Amsterdam 6. Amsterdam UMC, location VUmc, Amsterdam 7. Antoni van Leeuwenhoek, Amsterdam 8. Antonius Hospital, Sneek 9. Beatrixziekenhuis Rivas Zorggroep, Gorinchem 10. Bravis Hospital, Bergen op Zoom/Roosendaal 11. Canisius Wilhelmina Hospital (CWZ), Nijmegen 12. Deventer Hospital, Deventer 13. Diakonessenhuis Utrecht, Utrecht 14. Dijklander Hospital, Hoorn/Purmerend 15. Elisabeth-TweeSteden Hospital (ETZ), Tilburg 16. Flevo Hospital, Almere 17. Franciscus Gasthuis Vlietland, Rotterdam 18. Gelre Hospitals, Apeldoorn, Zutphen 19. Green Heart Hospital, Gouda 20. Haaglanden Medical Center, Den Haag 21. Hospital Gelderse Vallei, Ede 22. Hospital Group Twente, Hengelo, Almelo 23. Hospital St Jansdal, Harderwijk 24. IJsselland Hospital, Capelle aan den IJssel 25. Ikazia Hospital, Rotterdam 26. Isala Hospitals, Zwolle, Meppel, Kampen, Steenwijk, Heerde 27. LangeLand Hospital, Zoetermeer 28. Laurentius Hospital, Roermond 29. Leids University Medical Center, Leiden 30. Maastricht University Medical Center, Maastricht 31. Maasziekenhuis Pantein, Beugen 32. Meander Medical Center, Amersfoort 33. Medical Center Leeuwarden, Leeuwarden 34. Medisch Spectrum Twente, Enschede 35. Nij Smellinghe, Drachten 36. Onze Lieve Vrouwe Gasthuis, Amsterdam 37. Rijnstate Hospital, Arnhem 38. Rode Kruis Ziekenhuis, Beverwijk 39. Sint Jansgasthuis, Weert 40. Spaarne Gasthuis, Haarlem, Hoofddorp 41. Stichting ZorgSaam Zeeuws Vlaanderen, Terneuzen 42. Van Weel-Bethesda Hospital, Dirksland 43. VieCuri Medical Center, Venray 44. Zaans Medical Center, Zaandam 45. Zuyderland Medical Center, Heerlen, Sittard-Geleen |
| **Study coördinators** | **Drs. A.A.J. Grüter**  *Address*: Amsterdam UMC, location VUmc, Department of Surgery, De Boelelaan 1117, 1118, 1081 HV, Amsterdam  *Email*: [a.gruter@amsterdamumc.nl](mailto:a.gruter@amsterdamumc.nl) ([alexandergruter@hotmail.com](mailto:alexandergruter@hotmail.com))  *Telephone*: +31 645552015  **Dr. U.K. Coblijn**  *Address*: Amsterdam UMC, location VUmc, Department of Surgery, De Boelelaan 1117, 1118, 1081 HV, Amsterdam  *Email*: [u.coblijn@amsterdamumc.nl](mailto:u.coblijn@amsterdamumc.nl) ([usha.coblijn@gmail.com](mailto:usha.coblijn@gmail.com))  *Telephone*: +31 643469794 |
| **Sponsor** | Amsterdam University Medical Center, location VUmc, De Boelelaan 1117, 1118, 1081 HV Amsterdam, the Netherlands. |
| **Independent expert** | **Dr. S.S. Gisbertz**  *Address*: Amsterdam UMC, location VUmc, Department of Surgery, De Boelelaan 1117, 1118, 1081 HV, Amsterdam  *Email*: [s.s.gisbertz@amsterdamumc.nl](mailto:s.s.gisbertz@amsterdamumc.nl) |
| **Statistician** | Dr. B.I. Lissenberg-Witte  *Email:* [b.lissenberg@amsterdamumc.nl](mailto:b.lissenberg@amsterdamumc.nl) |

**TABLE OF CONTENTS**

1. LIST OF ABBREVIATIONS AND RELEVANT DEFINITIONS 8

2. SUMMARY 10

3. INTRODUCTION AND RATIONALE 12

4. OBJECTIVES AND HYPOTHESIS 13

4.1 Main study objective 13

4.2 Other study objectives 13

4.3 Hypothesis 13

5. STUDY DESIGN 15

6. STUDY POPULATION 16

6.1 Population (base) 16

6.2 Inclusion criteria 16

6.3 Exclusion criteria 16

6.4 Sample size calculation 16

7. TREATMENT OF SUBJECTS 18

7.1 Investigational treatment 18

8. METHODS 19

8.1 Study parameters/endpoints 19

8.1.1 Main study parameter/endpoint 19

8.1.2 Secondary study parameters/endpoints 19

8.2 Study procedures 19

8.3 Withdrawal of individual subjects 22

8.4 Follow-up of subjects withdrawn from treatment 22

9. SAFETY REPORTING 23

9.1 Temporary halt for reasons of subject safety 23

9.2 AEs, SAEs and SUSARs 23

9.2.1 Adverse events (AEs) 23

9.2.2 Serious adverse events (SAEs) 23

9.3 Follow-up of adverse events 24

9.4 Data Safety Monitoring Board (DSMB) 24

10. STATISTICAL ANALYSIS 25

11. ETHICAL CONSIDERATIONS 26

11.1 Regulation statement 26

11.2 Recruitment and consent 26

11.3 Objection by minors or incapacitated subjects (if applicable) 26

11.4 Benefits and risks assessment, group relatedness 26

11.5 Compensation for injury 26

11.6 Incentives (if applicable) 27

12. ADMINISTRATIVE ASPECTS, MONITORING AND PUBLICATION 28

12.1 Handling and storage of data and documents 28

12.2 Amendments 28

12.3 Annual progress report 29

12.4 Temporary halt and (prematurely) end of study report 29

12.5 Public disclosure and publication policy 29

13. APPENDIX 30

13.1 Study flowsheet showing patient pathway and CRF completion times 30

30

14. REFERENCES 31

# LIST OF ABBREVIATIONS AND RELEVANT DEFINITIONS

| **ABR** | General Assessment and Registration form (ABR form), the application form that is required for submission to the accredited Ethics Committee; in Dutch: Algemeen Beoordelings- en Registratieformulier (ABR-formulier) |
| --- | --- |
| **AE** | Adverse Event |
| **AR** | Adverse Reaction |
| **CA**  **CAT** | Competent Authority  Competency Assessment Tool |
| **CCMO**  **CME** | Central Committee on Research Involving Human Subjects; in Dutch: Centrale Commissie Mensgebonden Onderzoek  Complete mesocolic excision |
| **CV**  **DFS** | Curriculum Vitae  Disease free survival |
| **DSMB** | Data Safety Monitoring Board |
| **EU** | European Union |
| **EudraCT** | European drug regulatory affairs Clinical Trials |
| **GCP** | Good Clinical Practice |
| **GDPR** | General Data Protection Regulation; in Dutch: Algemene Verordening Gegevensbescherming (AVG) |
| **IB** | Investigator’s Brochure |
| **IC** | Informed Consent |
| **IMP** | Investigational Medicinal Product |
| **IMPD**  **IQR**  **LRHC** | Investigational Medicinal Product Dossier  Interquartile range  Laparoscopic Right Hemicolectomy |
| **METC**  **OS**  **PLCRC** | Medical research ethics committee (MREC); in Dutch: medisch-ethische toetsingscommissie (METC)  Overall survival  Prospective Dutch ColoRectal Cancer cohort; in Dutch: Prospectief Landelijk CRC cohort |
| **(S)AE**  **SD** | (Serious) Adverse Event  Standard deviation |
| **SPC** | Summary of Product Characteristics; in Dutch: officiële productinformatie IB1-tekst |
| **Sponsor** | The sponsor is the party that commissions the organisation or performance of the research, for example a pharmaceutical  company, academic hospital, scientific organisation or investigator. A party that provides funding for a study but does not commission it is not regarded as the sponsor, but referred to as a subsidising party. |
| **SUSAR** | Suspected Unexpected Serious Adverse Reaction |
| **UAVG** | Dutch Act on Implementation of the General Data Protection Regulation; in Dutch: Uitvoeringswet AVG |
| **WMO** | Medical Research Involving Human Subjects Act; in Dutch: Wet Medisch-wetenschappelijk Onderzoek met Mensen |

# SUMMARY

**Rationale:** Surgical procedures for gastrointestinal oncology intervention are inevitably highly variable amongst surgeons and centers. Although acceptable to a degree, a substantial proportion of this variability has a potential relevance for both short term clinical outcomes and long term survival. For patients with right-sided colon cancer, a laparoscopic right hemicolectomy (LRHC) is the surgical procedure to remove the cancer and locoregional lymph nodes. This surgical technique has evolved during the last decade with the introduction of the intracorporeal anastomosis, the Pfannenstiel extraction and the complete mesocolic excision (CME). The latter is a dissection technique in embryological planes with a central vascular ligation of the segmental branches at its origin, resulting in an optimal lymphadenectomy. Given the insights from recent studies showing a positive association between the quality of surgery and relevant clinical outcomes, there is a great need to reduce the interinstitutional and intersurgeon variability and to implement an optimized and standardized surgical technique for right-sided colon cancer in the Netherlands to improve short- and long-term clinical and oncological outcomes. This kind of implementation needs a consensus of the key elements of the procedure and a formative quality assessment of LRHC. Detailed objective assessment of the LRHC is currently not performed in clinical practice nor in surgical training. Quality assessment of LRHC has great potential to improve surgical training and furthermore, implementation of a standardized technique will ultimately lead to better quality of care for patients suffering from right-sided colon cancer.

**Objective**: The main objective of this study is to improve surgical outcomes for patients with right-sided colon cancer by a prospective sequential interventional cohort study that aims to standardize the surgical technique with subsequent controlled implementation after standardized review of the current practice in a nationwide multicenter setting.

**Study design:** Prospective interventional sequential cohort study.

**Study population:** Patients with planned laparoscopic or robot-assisted (extended) right hemicolectomy for colon cancer of the caecum, ascending colon or hepatic flexure.

**Intervention:** Implementation of the standardized right hemicolectomy with proctoring during a subsequent period with prospective inclusion of consecutive patients and the collection of surgical videos in all participating hospitals.

**Main study parameters/endpoints:** The primary endpoint is the 90-day morbidity according to the Clavien-Dindo classification system. The design will be a non-inferiority trial to ensure safety during the implementation of a new adapted surgical technique. Secondary endpoints include 90-day mortality, intraoperative complications (i.e. vascular injury), conversion rate, operative time, blood loss, validated assessment of the dissection plane, validated assessment of the level of vascular ligation, grading of the resection specimen, total lymph node count, number of resected positive lymph nodes, resection margins, completeness of mesocolic excision based on the one year postoperative CT imaging, locoregional recurrence, distant metastasis, three year disease free survival (DFS), five year overall survival (OS) and long term morbidity (incisional hernia, adhesion related small bowel obstruction, readmissions, reinterventions, anastomotic leakage).

# INTRODUCTION AND RATIONALE

Right-sided colon cancer, located in the caecum, ascending colon or hepatic flexure is most often treated with the laparoscopic (extended) right hemicolectomy (LRHC). The aim of this surgical procedure is to remove the right hemicolon with a radical resection of the tumor and an intact colic mesentery containing all draining lymph nodes, to restore bowel continuity, and to do so, with minimal morbidity. The quality of the LRHC is first of all determined by the correct plane of dissection and the extent of lymph node dissection according to oncological principles that provide an optimal locoregional control and staging information. Furthermore, the surgical quality can be reflected by patient relevant short- and long-term clinical outcomes [1-3].

Unlike treatment with medication, a surgical intervention might be highly variable amongst surgeons and centers. This variability has a potential relevance concerning the clinical and oncological outcomes. As can be expected, it is known that the learning curve of surgeons adapting a new technique without standardized training and an implementation program can result in adverse clinical outcomes, such as a higher conversion and complication rate [4]. Therefore, standardized surgical training and proctoring of a new surgical technique is associated with a decrease of the negative impact caused by the learning curve [5]. Additionally, standardization of surgical techniques and credentialing of surgeons were found to reduce the adjusted in-hospital mortality [6, 7]. Recently, a large cohort study identified that good technical surgical skills are associated with a better long-term survival of patients with colorectal cancer [8].

For right-sided colon cancer, substantial procedural variation is present [9, 10]. The most important changes to the LRHC techniques in the last decade are the intracorporeal anastomosis and the complete mesocolic excision (CME) technique. CME encompasses a dissection along embryological planes to create an intact envelope of mesocolic fascia with a central vascular ligation to include all the lymph nodes along the supplying segmental vessels [11-14]. A questionnaire amongst trainees in the Netherlands confirmed the presence of a high variability in the surgical technique for right hemicolectomy. There is a great need for a formative quality assessment of laparoscopic right hemicolectomy, given the insights from recent studies showing the association between quality of surgery and relevant clinical outcomes. Detailed objective assessment of the laparoscopic right hemicolectomy is currently not done in clinical practice nor in surgical training. Quality assessment of laparoscopic right hemicolectomy has great potential to improve surgeon training and implementation of a standardized technique will ultimately lead to better quality of care for patients with colon cancer.

# OBJECTIVES AND HYPOTHESIS

## Main study objective

The aim of this study proposal is to improve surgical outcomes for patients with right-sided colon cancer by a prospective sequential interventional cohort study that aims to standardize the laparoscopic right hemicolectomy technique with subsequent controlled implementation after standardized review of current practice in a nationwide multicenter setting.

## Other study objectives

- To evaluate current practice and variations in technique for laparoscopic right hemicolectomy in the Netherlands.

- To give an overview of the vascular anatomical variations pertinent to laparoscopic right hemicolectomy.

- To evaluate the association of each variation performed in laparoscopic right hemicolectomy with the short- and long-term clinical and oncological outcomes.

- To reach consensus for a standardized step wise technique for laparoscopic right hemicolectomy using the most recent insights in optimized colon cancer surgery from literature and the results from the assessment during the first phase of the study, according to the Delphi method.

- Development and validation of a Competency Assessment Tool (CAT) for the standardized right hemicolectomy using surgical full-length videos of the laparoscopic right hemicolectomy procedures and the steps as identified in the Delphi method.

- To train and implement the standardized surgical technique in centers in the Netherlands for patients with right-sided colon cancer.

- To evaluate the short- and long-term clinical and oncological outcomes of the cohort after training and compare these with the cohort before training, implementation and proctoring of the standardized technique.

## Hypothesis

The primary hypothesis of this study is that the implemented and standardized laparoscopic surgical technique for right-sided colon cancer will be safe without an increase in the 90 days postoperative morbidity (non-inferiority design).

The second hypothesis is that the quality of surgery, objectively assessed by competency assessment, is an important determinant for long-term clinical patient outcomes.

The clinical outcomes of the surgical technique to treat right-sided colon cancer will be improved by safely implementing a standardized method according to the latest evidence. Improvements in outcome measures that reflect the oncological quality of surgery, such as validated assessment of plane of dissection and vascular ligation, grading of the resection specimen, total lymph node count and number of resected positive lymph nodes and completeness of mesocolic excision based on postoperative CT imaging are expected. Eventually, it is hypothesized that the surrogate oncological endpoints will translate in reduction of local and distant recurrences and improved 5-year overall survival (OS) and 3-year disease free survival (DFS).

# STUDY DESIGN

The design of this trial is a multicenter prospective interventional sequential cohort study with a transition period in which implementation of the standardized technique is performed in the same centers after education and hands on training. Proctoring of the new technique will be provided during implementation. The second data evaluation of the clinical and oncological outcomes of the consecutive patient group will be collected in the group of patients after proctoring is finished, the so called consolidation phase. This study is primarily designed as a non-inferiority trial, based on short-term postoperative complications, with the aim of ultimate improvement of long-term oncological outcome. The accrual of both the control patients and the patients after implementation will be within the national Prospective Dutch ColoRectal Cancer cohort (PLCRC) with additional video analysis and image collection with additional informed consent. The primary endpoint is safety during implementation of a new technique. This is measured by 90-day morbidity with the Clavien-Dindo classification [15]. The assumption is that the perioperative morbidity (26% 30-day overall postoperative complication rate; as earlier published based on the DCRA database [16]) will not be increased by implementation of the new standardized technique with a non-inferiority margin of 7%, therefore not exceeding 33%.

# STUDY POPULATION

## Population (base)

Patients with a planned laparoscopic or robot-assisted (extended) right hemicolectomy for colon cancer.

## Inclusion criteria

In order to be eligible to participate in this study, a subject must meet all of the following criteria:

- Planned laparoscopic or robot-assisted (extended) right hemicolectomy for colon cancer of the caecum, ascending colon, hepatic flexure or proximal transverse colon;
- Age above 18 years;
- Signed informed consent.

## Exclusion criteria

A potential subject who meets any of the following criteria will be excluded from participation in this study:

- cT4b/multivisceral resection;
- cTNM stage 4 (M1);
- ASA 4;
- Immune modulating medication;
- Prior midline or transverse laparotomy larger than 10 cm (not including Pfannenstiel and McBurney's incision);
- Perforated disease/peritumoral abscess/fistula;
- Acute obstruction;
- Emergency surgery;
- Neuroendocrine neoplasm (NEN);
- Other primary malignancy treated within 5 years from diagnosis of colon cancer, except for curatively treated prostate, breast, skin and cervical cancer.

## Sample size calculation

Sample size calculation is performed with the aim to demonstrate safety with non-inferiority for the primary endpoint overall 90-day morbidity. If there is truly no difference between the standard and experimental treatment, then 1095 patients (365 patient in each of the three groups) are required to be 80% sure that the lower limit of a one-sided 95% confidence interval (or equivalently a 90% two-sided confidence interval) will be above the non-inferiority limit of 7%. Therefore, in the one group 365 patients will be included (control group) and in the other group 730 patients will be included (implementation and consolidation phase together).

# TREATMENT OF SUBJECTS

## Investigational treatment

The implementation of the standardized laparoscopic right hemicolectomy with proctoring with prospective inclusion of consecutive patients with collection of surgical videos in all participating hospitals will be investigated. This method will be compared with the laparoscopic right hemicolectomy performed by the same hospitals before the training and implementation of the standardized technique. The clinical outcomes will be collected using the DCRA database and the oncological outcomes will be collected by the surgeons, surgical registrars and/or trained research nurses in the participating centers.

# METHODS

## Study parameters/endpoints

### Main study parameter/endpoint

The primary endpoint is the 90-day morbidity with Clavien-Dindo classification.

### Secondary study parameters/endpoints

- Intraoperative complications (i.e. vascular injury, injury to other organs);
- 90-day mortality;
- Conversion;
- Operative time;
- Blood loss;
- Validated assessment of plane of dissection;
- Validated assessment of level of vascular ligation;
- Grading of the resection specimen according to Benz et al. [17];
- Total lymph node count;
- Number of resected positive lymph nodes;
- Resection margins;
- Completeness of mesocolic excision based on postoperative CT imaging;
- Locoregional recurrence;
- Distant metastasis;
- 3-year disease free survival (DFS);
- 5-year overall survival (OS);
- Long-term morbidity: incisional hernia, adhesion related small bowel obstruction, readmissions, reinterventions, anastomotic leakage.

## Study procedures

First the variability of surgical techniques amongst surgeons and centers for right-sided colon cancer will be evaluated by a 4 months period (possibility to extend until maximum of 6 months) prospective inclusion of 365 patients and evaluating preoperative CT imaging, full length surgical video and clinicopathological outcomes, as well as the CT imaging one year post-operatively as routine part of oncological follow-up.

Second, a Delphi method amongst participating colorectal surgeons will be applied after the 4 months period (possibility to extend until maximum of 6 months) of video collection, to establish a detailed standardized technique for laparoscopic right hemicolectomy for colon cancer. This technique will include essential elements that can potentially improve outcomes such as approach, number and positioning of trocars, plane of retroperitoneal dissection, level of vascular ligation with extent of vertical lymphadenectomy, technique for constructing the anastomosis and preferred extraction site.

Third, the standardized technique will be taught during hands-on surgical training.

Fourth, the new technique will be implemented in a controlled fashion with proctoring in the same centers, with another 4 months period (possibility to extend until maximum of 6 months) of prospective inclusion of 365 patients.

Finally, a third consecutive group of patients will be included to consolidate the standardized technique after the learning curve. The same preoperative CT imaging, full length surgical videos and clinicopathological outcomes as well as the CT imaging one year post surgery will be collected and analyzed, in order to be able to compare the performance with the pre-implementation period. Again, 365 patients will be prospectively included in a 4 months period (possibility to extend until maximum of 6 months).

Schematic five step approach:


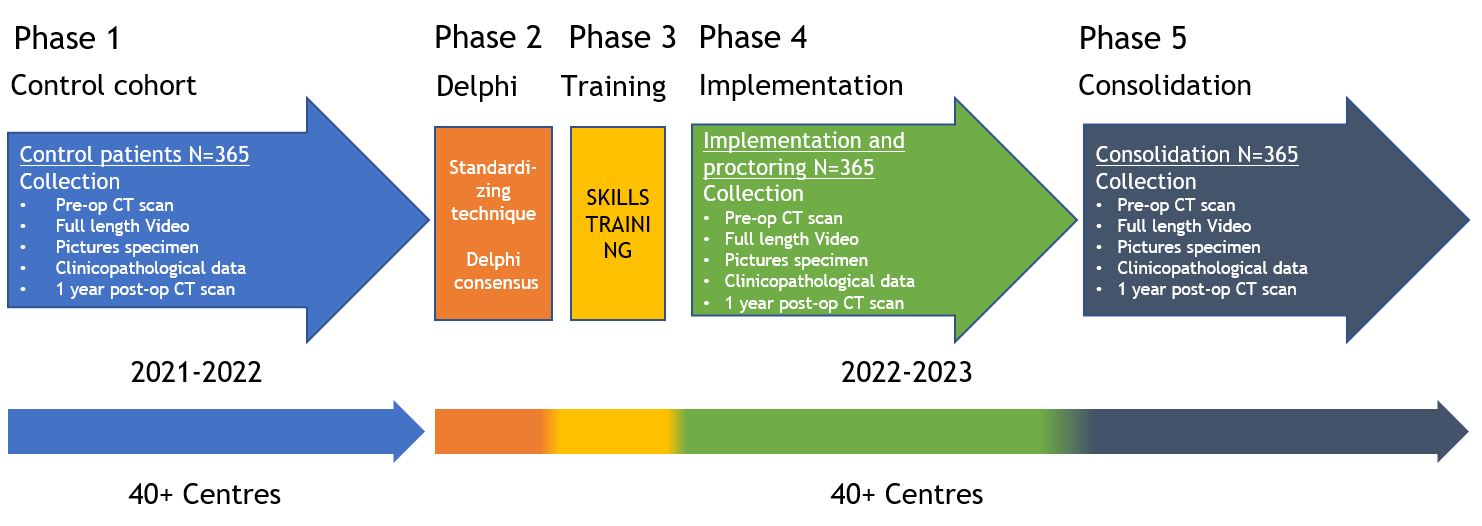


1. **Control cohort phase**: Prospective mapping of current practice with surgical variations in laparoscopic right hemicolectomy (total duration of inclusion 4 months, possibility to extend until maximum of 6 months). N=40+ centers; N=365 videos
   1. METC approval for the use of anonymized videos of a laparoscopic procedure and collection of corresponding clinical outcomes.
   2. Approaching hospitals for participation (high volume centers (50+ colon cancers).
   3. Prospective inclusion of consecutive patients undergoing laparoscopic right hemicolectomy in the participating hospitals in three months.
2. **Delphi phase**: Development of Standard Laparoscopic Right Hemicolectomy: an (inter)national Delphi study
   1. Approaching specialists to participate in the Delphi method (from participating hospitals in step 1).
   2. Identification of crucial steps and measures according to literature and expert’s opinion (Delphi method).
   3. Documentation of steps and the order in which they need to be performed, development of a competency assessment tool (CAT).
3. **Training phase**: Skills center training facility
   1. Training the participating surgeons in the method of the standardized laparoscopic right hemicolectomy, as consented in the Delphi method.
4. **Implementation phase**: implementing the standardized laparoscopic right hemicolectomy with proctoring during another period with prospective inclusion of 365 consecutive patients (total duration of inclusion is 4 months, possibility to extend until maximum of 6 months). N=40+ centers; N=365 videos.
5. **Consolidation phase**: Collection of a third group of 365 consecutive patients with collection of surgical videos in all participating hospitals (total duration of inclusion is 4 months, possibility to extend until maximum of 6 months). N=40+ centers; N=365 videos.
   1. Rating videos.
   2. Comparing with pre-implementation performance based on outcome measures reflecting oncological quality of surgery, and 90-day clinical outcomes.
   3. Comparing with pre-implementation performance based on the long-term outcomes. (3-year DFS and 5-year OS).

## Withdrawal of individual subjects

Subjects can leave the study at any time for any reason if they wish to do so without any consequences. The investigator can decide to withdraw a subject from the study for urgent medical reasons.

## Follow-up of subjects withdrawn from treatment

Patients whom have withdrawn from the study, but are still willing in participating in the follow-up will be followed according to the specifications of the patient.

# SAFETY REPORTING

## Temporary halt for reasons of subject safety

In accordance to section 10, subsection 4, of the WMO, the sponsor will suspend the study if there is sufficient ground that continuation of the study will jeopardise subject health or safety. The sponsor will notify the accredited METC without undue delay of a temporary halt including the reason for such an action. The study will be suspended pending a further positive decision by the accredited METC. The investigator will take care that all subjects are kept informed.

## AEs, SAEs and SUSARs

### Adverse events (AEs)

Adverse events are defined as any undesirable experience occurring to a subject during the study, whether or not considered related to the investigational treatment. All adverse events reported spontaneously by the subject or observed by the investiga­tor or his staff will be recorded.

### Serious adverse events (SAEs)

A serious adverse event is any untoward medical occurrence or effect that

- results in death;
- is life threatening (at the time of the event);
- requires hospitalisation or prolongation of existing inpatients’ hospitalisation;
- results in persistent or significant disability or incapacity;
- is a congenital anomaly or birth defect; or
- any other important medical event that did not result in any of the outcomes listed above due to medical or surgical intervention but could have been based upon appropriate judgement by the investigator.

An elective hospital admission will not be considered as a serious adverse event.

The sponsor will report the SAEs through the web portal *ToetsingOnline* to the accredited METC that approved the protocol, within 7 days of first knowledge for SAEs that result in death or are life threatening followed by a period of maximum of 8 days to complete the initial preliminary report. All other SAEs will be reported within a period of maximum 15 days after the sponsor has first knowledge of the serious adverse events.

## Follow-up of adverse events

All AEs will be followed until they have abated, or until a stable situation has been reached. Depending on the event, follow up may require additional tests or medical procedures as indicated, and/or referral to the general physician or a medical specialist.

SAEs need to be reported till end of study within the Netherlands, as defined in the protocol.

## Data Safety Monitoring Board (DSMB)

A DSMB will be assigned.

# STATISTICAL ANALYSIS

The clinical outcomes will be categorical or dichotomous outcomes and will be presented as absolute numbers and percentages. The chi-square test will be used for comparing categorical data among patient groups. Descriptive outcomes will be reported as median with interquartile range (IQR) or mean with standard deviation (SD) and in accordance to their distribution the Mann-Whitney-U test is used to evaluate intergroup variation.

Oncological outcome will be determined using Kaplan-Meier analyses, with comparison of relevant patient groups using the log-rank test. Predictors of main outcome parameters will be determined by selecting relevant variables based on expected association with subsequent univariable analysis. Besides stratified comparisons as described above, multivariable analysis will be performed to determine independent association of factors with a specific outcome parameter using logistic and Cox regression analyses. A p-value <0.05 will be considered to be statistically significant. All analyses will be performed with IBM SPSS statistics, version 23.00 (IBM Corp Amonk, NY, United States).

# ETHICAL CONSIDERATIONS

## Regulation statement

This study will be conducted according to the principles of the Declaration of Helsinki (Fortaleza, Brasil, October 2013) and in accordance with the Medical Research Involving Human Subjects Act (WMO) and other guidelines, regulations and acts.

## Recruitment and consent

Suitable patients will be approached for entry into the trial at the first outpatient visit at the surgery department after diagnosis of colon cancer of the caecum, ascending colon or hepatic flexure has been made. The rationale for the trial is explained to the patient. A written patient information sheet is provided and patients will be given the opportunity to ask questions. The informed consent will be obtained before the operation. Written informed consent is taken by surgeons, surgical registrars or trained research nurses. When consent has been obtained, the original form is kept in the trial file and a copy is given to the patient. Baseline data as well as baseline questionnaires are collected.

## Objection by minors or incapacitated subjects (if applicable)

Minors and legally incompetent adults are excluded from the trial.

## Benefits and risks assessment, group relatedness

Patients included in the first period (prospective mapping of surgical variations) do not directly benefit from participation in this study. Patients included in the second period (implementation of standardized laparoscopic right hemicolectomy) might benefit from the implementation and could have less postoperative complications.

## Compensation for injury

The sponsor/investigator has a liability insurance which is in accordance with article 7 of the WMO.

The sponsor (also) has an insurance which is in accordance with the legal requirements in the Netherlands (Article 7 WMO). This insurance provides cover for damage to research subjects through injury or death caused by the study.

The insurance applies to the damage that becomes apparent during the study or within 4 years after the end of the study.

## Incentives (if applicable)

Enrolled patients will not receive any special interventions, compensation or treatment through participation in this trial.

# ADMINISTRATIVE ASPECTS, MONITORING AND PUBLICATION

## Handling and storage of data and documents

Every included patient will be assigned a three digit study number. Communication occurs only with this number. The full name and date of birth of the patient will only be recorded on the informed consent form.

A study coordinator coordinates the study, monitors patient inclusion and protocol steps, data collection, data entry, preparation and performs analyses and will report the data. Continuous data monitoring and data collection on a CRF will guarantee complete and real-time prospective recording of data. Data will be collected and stored at the VUmc in a separate, closed room.

There is a collaboration with FormsVision, a privately owned business delivering clinical IT solutions to the academic community and the pharmaceutical industry, that can build the digital platform (<https://www.aleaclinical.eu/>) to facilitate assessment of high volume full length videos of surgical procedures with clinical safety requirements concerning privacy regulations. The design allows secure online worldwide access to upload unlimited data 10+ GB full length video and allows automatic consignments to reviewers by email and link towards reviewer account with the ability to assess the quality of surgery. The digital platform is also designed to add all relevant clinical data related to clinical outcomes.

## Amendments

Amendments are changes made to the research after a favourable opinion by the accredited METC has been given. All amendments will be notified to the METC that gave a favourable opinion.

All substantial amendments will be notified to the METC and to the competent authority.

Non-substantial amendments will not be notified to the accredited METC and the competent authority, but will be recorded and filed by the sponsor.

A ‘substantial amendment’ is defined as an amendment to the terms of the METC application, or to the protocol or any other supporting documentation, that is likely to affect to a significant degree:

- The safety or physical or mental integrity of the subjects of the trial;
- The scientific value of the trial;
- The conduct or management of the trial; or
- The quality or safety of any intervention used in the trial.

## Annual progress report

The sponsor/investigator will submit a summary of the progress of the trial to the accredited METC once a year. Information will be provided on the date of inclusion of the first subject, numbers of subjects included and numbers of subjects that have completed the trial, serious adverse events/ serious adverse reactions, other problems, and amendments.

## Temporary halt and (prematurely) end of study report

The investigator/sponsor will notify the accredited METC of the end of the study within a period of 8 weeks. The end of the study is defined as the last patient’s last visit.

The sponsor will notify the METC immediately of a temporary halt of the study, including the reason of such an action.

In case the study is ended prematurely, the sponsor will notify the accredited METC within 15 days, including the reasons for the premature termination.
Within one year after the end of the study, the investigator/sponsor will submit a final study report with the results of the study, including any publications/abstracts of the study, to the accredited METC.

## Public disclosure and publication policy

Patients are entitled to public disclosure of the results of the trial on the basis of their participation in it. The results of research will be submitted for publication to peer-reviewed scientific journals. Agreements with respect to participation in publication were made before the start of the trial. Authorship is granted to all people of the study group. Every other people who made substantial contribution to the trial will be added to the collaborator list.

# APPENDIX

## Study flowsheet showing patient pathway and CRF completion times

Patient pathway: Suggested CRF completion times:

Decision to operate

CRF 1: Registration form

CRF 2: Patient characteristics

CRF 3: Preoperative CT-imaging

CRF 4: Preoperative tumor characteristics

CRF 5: Video and surgical details

CRF 6: Specimen

CRF 7: Pathology

CRF 8: Follow-up 3 months

CRF 9: Follow-up 12 months

CRF 10: 1 year postoperative CT-imaging

CRF 11: Follow-up 36 months

CRF 12: Follow-up 60 months

CRF 7-12

CRF 4-6

CRF 1-3

Patient discharged

Operation

Pre-operative assessment clinic

Routine outpatient review

(around 4-6 weeks)

# REFERENCES

1. Birkmeyer, J.D., et al., *Surgical skill and complication rates after bariatric surgery.* N Engl J Med, 2013. **369**(15): p. 1434-42.

2. Curtis, N.J., et al., *Association of Surgical Skill Assessment With Clinical Outcomes in Cancer Surgery.* JAMA Surg, 2020. **155**(7): p. 590-598.

3. Stulberg, J.J., et al., *Association Between Surgeon Technical Skills and Patient Outcomes.* JAMA Surg, 2020.

4. Miskovic, D., et al., *Learning curve and case selection in laparoscopic colorectal surgery: systematic review and international multicenter analysis of 4852 cases.* Dis Colon Rectum, 2012. **55**(12): p. 1300-10.

5. Torricelli, F.C., J.A. Barbosa, and G.S. Marchini, *Impact of laparoscopic surgery training laboratory on surgeon's performance.* World J Gastrointest Surg, 2016. **8**(11): p. 735-743.

6. Markar, S.R., et al., *Assessment of the quality of surgery within randomised controlled trials for the treatment of gastro-oesophageal cancer: a systematic review.* Lancet Oncol, 2015. **16**(1): p. e23-31.

7. Hanna, G.B., et al., *Laparoscopic Colorectal Surgery Outcomes Improved After National Training Program (LAPCO) for Specialists in England.* Ann Surg, 2020.

8. Brajcich, B.C., et al., *Association Between Surgical Technical Skill and Long-term Survival for Colon Cancer.* JAMA Oncol, 2021. **7**(1): p. 127-129.

9. Li, F., et al., *Comparison between different approaches applied in laparoscopic right hemi-colectomy: A systematic review and network meta-analysis.* International Journal of Surgery, 2017. **48**: p. 74-82.

10. Matsuda, T., et al., *Current status and trend of laparoscopic right hemicolectomy for colon cancer.* Ann Gastroenterol Surg, 2020. **4**(5): p. 521-527.

11. Munkedal, D.L.E., et al., *Radiological and pathological evaluation of the level of arterial division after colon cancer surgery.* Colorectal Dis, 2017. **19**(7): p. O238-O245.

12. Sondenaa, K., et al., *The rationale behind complete mesocolic excision (CME) and a central vascular ligation for colon cancer in open and laparoscopic surgery : proceedings of a consensus conference.* Int J Colorectal Dis, 2014. **29**(4): p. 419-28.

13. van Oostendorp, S., et al., *Intracorporeal versus extracorporeal anastomosis in right hemicolectomy: a systematic review and meta-analysis.* Surg Endosc. **31**(1): p. 64-77.

14. Enomoto, M., et al., *Laparoscopic complete mesocolic excision with true central vascular ligation for right-sided colon cancer.* Surg Endosc.

15. Clavien, P.A., et al., *The Clavien-Dindo classification of surgical complications: five-year experience.* Ann Surg, 2009. **250**(2): p. 187-96.

16. Bosker, R.J.I., et al., *Minimally Invasive versus Open Approach for Right-Sided Colectomy: A Study in 12,006 Patients from the Dutch Surgical Colorectal Audit.* Dig Surg, 2019. **36**(1): p. 27-32.

17. Benz, S., et al., *Proposal of a new classification system for complete mesocolic excison in right-sided colon cancer.* Tech Coloproctol, 2019. **23**(3): p. 251-257.
