# Supplementary material for: Nationwide variations in the execution of minimally invasive right hemicolectomy and short-term outcomes: first phase of the RIGHT study
Source: Br J Surg. 2024 Nov 18;111(11):znae291. doi: 10.1093/bjs/znae291 (PMC11572717; doi:10.1093/bjs/znae291)
Supplement: znae291_Supplementary_Data [file znae291_supplementary_data.zip › Supplementary_File_2._Original_study_protocol_phase_1_Right_study.docx]

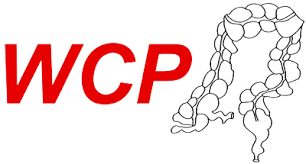


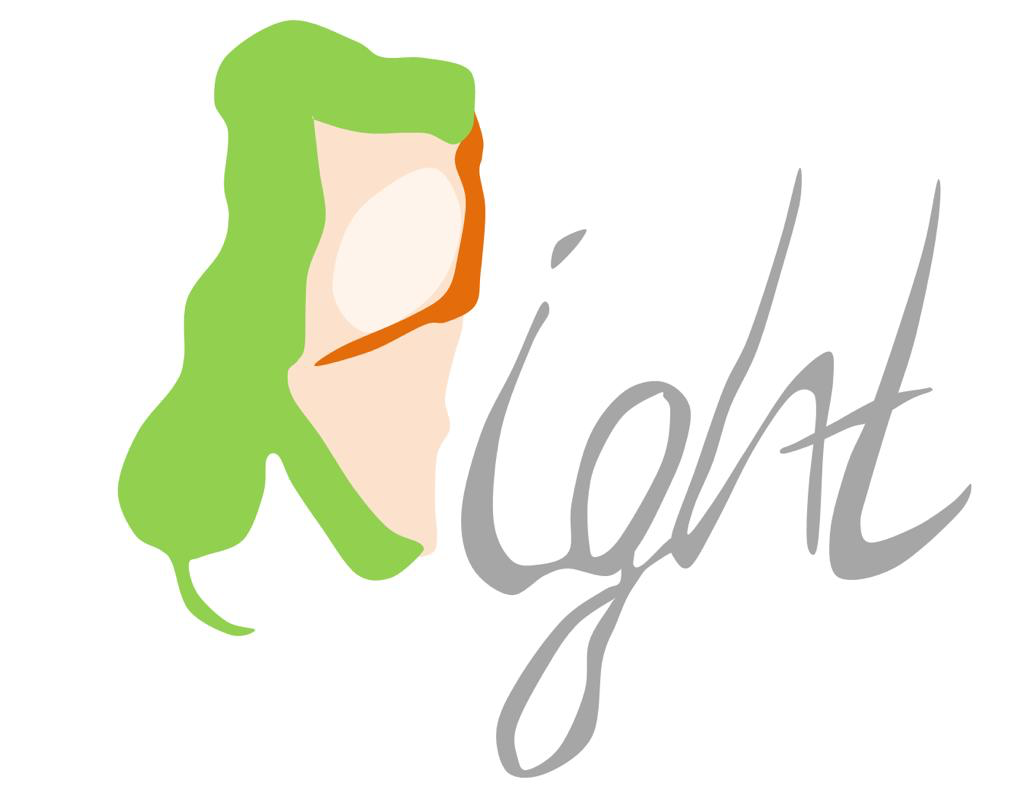


STUDY

**(voor aanvraag niet-WMO verklaring)**

Onderzoeksprotocol Phase 1

**Algemene gegevens**

| **Titel** | Prospective mapping of surgical variations in laparoscopic right hemicolectomy and development of optimized and standardized surgical technique for right-sided colon cancer |
| --- | --- |
| **Datum** | 29-06-2021 |
| **Versienummer** | 3 |
| **Indiener** | A.A.J. Grüter |
| **Coördinerende onderzoeker** | **Drs. A.A.J. Grüter**  *Address*: Amsterdam UMC, location VUmc, Department of Surgery, De Boelelaan 1117, 1118, 1081 HV, Amsterdam  *Email*: [a.gruter@amsterdamumc.nl](mailto:a.gruter@amsterdamumc.nl)  *Telephone*: +31 645552015  **Dr. U.K. Coblijn**  *Address*: Amsterdam UMC, location VUmc, Department of Surgery, De Boelelaan 1117, 1118, 1081 HV, Amsterdam  *Email*: [usha.coblijn@gmail.com](mailto:usha.coblijn@gmail.com)  *Telephone*: +31 643469794 |
| **Hoofdonderzoeker(s)** | **Dr. J.B. Tuynman, project leader**  *Address*: Amsterdam UMC, location VUmc, Department of Surgery, De Boelelaan 1117, 1118, 1081 HV, Amsterdam  *Email*: [j.tuynman@amsterdamumc.nl](mailto:j.tuynman@amsterdamumc.nl)  *Telephone*: +31 630155307  **Dr. B.R. Toorenvliet**  *Address*: Ikazia Hospital Rotterdam, Department of Surgery, Montessoriweg 1, 3083 AN, Rotterdam  *Email*: [br.toorenvliet@ikazia.nl](mailto:br.toorenvliet@ikazia.nl)  Telephone: +31 650251180  **Prof. dr. P.J. Tanis**  *Address*: Amsterdam UMC, location VUmc, Department of Surgery, De Boelelaan 1117, 1118, 1081 HV, Amsterdam  *Email*: [p.j.tanis@amsterdamumc.nl](mailto:p.j.tanis@amsterdamumc.nl)  *Telephone*: +31 629068275 |
| **Opdrachtgever (verrichter)** | Amsterdam UMC, location VUmc |

**Onderzoekgegevens**

| **Rationale** | Right-sided colon cancer, located in the caecum, ascending colon or hepatic flexure is most often treated with the laparoscopic (extended) right hemicolectomy (LRHC). The aim of this surgical procedure is to remove the right hemicolon with a radical resection of the tumor and an intact colic mesentery containing all draining lymph nodes, to restore bowel continuity, and to do so, with minimal morbidity. The quality of the LRHC is first of all determined by the correct plane of dissection and the extent of lymph node dissection according to oncological principles that provide an optimal locoregional control and staging information. Furthermore, the surgical quality can be reflected by patient relevant short- and long-term clinical outcomes.  Unlike treatment with medication, a surgical intervention might be highly variable amongst surgeons and centers. This variability has a potential relevance concerning the clinical and oncological outcomes. As can be expected, it is known that the learning curve of surgeons adapting a new technique without standardized training and an implementation program can result in adverse clinical outcomes, such as a higher conversion and complication rate. Therefore, standardized surgical training and proctoring of a new surgical technique is associated with a decrease of the negative impact caused by the learning curve. Additionally, standardization of surgical techniques and credentialing of surgeons were found to reduce the adjusted in-hospital mortality. Recently, a large cohort study identified that good technical surgical skills are associated with a better long term survival of patients with colorectal cancer.  For right-sided colon cancer, substantial procedural variation is present. The most important changes to the LRHC techniques in the last decade are the intracorporeal anastomosis and the complete mesocolic excision (CME) technique. CME encompasses a dissection along embryological planes to create an intact envelope of mesocolic fascia with a central vascular ligation to include all the lymph nodes along the supplying segmental vessels. A questionnaire amongst trainees in the Netherlands confirmed the presence of a high variability in the surgical technique for right hemicolectomy. There is a great need for a formative quality assessment of laparoscopic right hemicolectomy, given the insights from recent studies showing the association between quality of surgery and relevant clinical outcomes. Detailed objective assessment of the laparoscopic right hemicolectomy is currently not done in clinical practice nor in surgical training. Quality assessment of laparoscopic right hemicolectomy has great potential to improve surgeon training and implementation of a standardized technique will ultimately lead to better quality of care for patients with colon cancer. |
| --- | --- |
| **Doel** | First the variability of surgical techniques amongst surgeons and centers for right-sided colon cancer will be evaluated by a 4 months prospective inclusion of patients and evaluating preoperative CT imaging, full length surgical video and clinic pathological outcomes, as well as the CT imaging one year post-operatively as routine part of oncological follow-up.  Second, a Delphi method amongst participating colorectal surgeons will be applied after the 3 months period of video collection, to establish a detailed standardized technique for laparoscopic right hemicolectomy for colon cancer. This technique will include essential elements that can potentially improve outcomes such as approach, number and positioning of trocars, plane of retroperitoneal dissection, level of vascular ligation with extent of vertical lymphadenectomy, technique for constructing the anastomosis and preferred extraction site. |
| **Studie design** | Prospective cohort study and Delphi study. |
| **Studie populatie** | Patients with a planned laparoscopic or robot-assisted (extended) right hemicolectomy for colon cancer. |
| **Inclusiecriteria** | - Planned laparoscopic or robot-assisted (extended) right hemicolectomy for colon cancer of the caecum, ascending colon, hepatic flexure or proximal transverse colon; - Age above 18 years; - Signed informed consent. |
| **Exclusiecriteria** | - cT4b/multivisceral resection; - cTNM stage 4 (M1); - ASA 4; - Immune modulating medication; - Prior midline or transverse laparotomy; - Perforated disease/peritumoral abcess/fistula; - Acute obstruction; - Emergency surgery; - Appendiceal cancer; - Other primary malignancy treated within 5 years from diagnosis of colon cancer, except for curatively treated prostate, breast, skin and cervical cancer. |
| **Aantal proefpersonen/ sample grootte** | Sample size calculation is performed with the aim to demonstrate safety with non-inferiority for the primary endpoint overall 30-day morbidity. If there is truly no difference between the standard (control cohort) and experimental treatment (later study), then 1095 patients (365 patient in each of the three groups) are required to be 80% sure that the lower limit of a one-sided 95% confidence interval (or equivalently a 90% two-sided confidence interval) will be above the non-inferiority limit of 7%.  Therefore, in the this study (control cohort) **365 patients** will be included. |
| **Werving proefpersonen** | Suitable patients will be approached for entry into the trial at the first outpatient visit at the surgery department after diagnosis of colon cancer of the caecum, ascending colon or hepatic flexure has been made. The rationale for the trial is explained to the patient. A written patient information sheet is provided and patients will be given the opportunity to ask questions. The informed consent will be obtained before the operation. Written informed consent is taken by surgeons, surgical registrars or trained research nurses. When consent has been obtained, the original form is kept in the trial file and a copy is given to the patient. Baseline data as well as baseline questionnaires are collected. |
| **Interventie** | Not applicable in this part of the study (control cohort). |
| **Standaardzorg /**  **Standaardbehandeling** | Not applicable in this part of the study (control cohort). |
| **Studie parameters** | The primary endpoint is the 30-day morbidity with Clavien-Dindo classification. |
| **Studie eindpunten** | - Intraoperative complications (i.e. vascular injury, injury to other organs); - 30-day mortality; - Conversion; - Operative time; - Blood loss; - Validated assessment of plane of dissection; - Validated assessment of level of vascular ligation; - Grading of the resection specimen according to Benz et al. [17]; - Total lymph node count; - Number of resected positive lymph nodes; - Resection margins; - Completeness of mesocolic excision based on postoperative CT imaging; - Locoregional recurrence; - Distant metastasis; - 3-year disease free survival (DFS); - 5-year overall survival (OS); - Long term morbidity: incisional hernia, adhesion related small bowel obstruction, readmissions, reinterventions, anastomotic leakage. |
| **Statistische analyses** | The clinical outcomes will be categorical or dichotomous outcomes and will be presented as absolute numbers and percentages. The chi-square test will be used for comparing categorical data among patient groups (this study versus the next study). Descriptive outcomes will be reported as median with interquartile range (IQR) or mean with standard deviation (SD) and in accordance to their distribution the Mann-Whitney-U test is used to evaluate intergroup variation.  Oncological outcome will be determined using Kaplan-Meier analyses, with comparison of relevant patient groups using the log-rank test. Predictors of main outcome parameters will be determined by selecting relevant variables based on expected association with subsequent univariable analysis. Besides stratified comparisons as described above, multivariable analysis will be performed to determine independent association of factors with a specific outcome parameter using logistic and Cox regression analyses. A p-value <0.05 will be considered to be statistically significant. All analyses will be performed with IBM SPSS statistics, version 23.00 (IBM Corp Amonk, NY, United States). |
| **Belasting voor de proefpersoon** | Not applicable. |
| **Risico voor de proefpersoon** | Not applicable. |
| **Voordelen deelname aan het onderzoek** | Not applicable. |
| **Nadelen deelname aan het onderzoek** | Not applicable. |
| **Vergoeding voor de proefpersoon** | Not applicable. |
| **Administratieve aspecten** | Every included patient will be assigned a three digit study number. Communication occurs only with this number. The full name and date of birth of the patient will only be recorded on the informed consent form.  A study coordinator coordinates the study, monitors patient inclusion and protocol steps, data collection, data entry, preparation and performs analyses and will report the data. Continuous data monitoring and data collection on a CRF will guarantee complete and real-time prospective recording of data. Data will be collected and stored at the VUmc in a separate, closed room.  There is a collaboration with FormsVision, a privately owned business delivering clinical IT solutions to the academic community and the pharmaceutical industry, that can build the digital platform (https://www.aleaclinical.eu/) to facilitate assessment of high volume full length videos of surgical procedures with clinical safety requirements concerning privacy regulations. The design allows secure online worldwide access to upload unlimited data 10+ GB full length video and allows automatic consignments to reviewers by email and link towards reviewer account with the ability to assess the quality of surgery. The digital platform is also designed to add all relevant clinical data related to clinical outcomes. |
| **Publicatiebeleid en amendementen** | The manuscripts will be submitted to an international peer reviewed journal. |
| **Overige punten van belang voor de METc** | - |
