# Supplementary material for: Nationwide variations in the execution of minimally invasive right hemicolectomy and short-term outcomes: first phase of the RIGHT study
Source: Br J Surg. 2024 Nov 18;111(11):znae291. doi: 10.1093/bjs/znae291 (PMC11572717; doi:10.1093/bjs/znae291)
Supplement: znae291_Supplementary_Data [file znae291_supplementary_data.zip › Supplementary_material.docx]

**Nationwide variations in the execution of minimally invasive right hemicolectomy and short-term outcomes; first phase of the Right study**

Alexander A.J. Grüter, MD^1,2^, Willemijn A. Jongsma, MD^1,2^, Nicola Leone, MD^1,3^, Hasti Barai^1^, Boudewijn R. Toorenvliet, MD, PhD^4^, Pieter J. Tanis, MD, PhD, Professor^5,6^, Jurriaan B. Tuynman, MD, PhD, Professor^1^, on behalf of the RIGHT collaborators group.

^1^Amsterdam UMC location Vrije Universiteit Amsterdam, Department of Surgery, De Boelelaan 1117, Amsterdam, The Netherlands.

^2^Cancer Center Amsterdam, Treatment and quality of life, Amsterdam, The Netherlands.

^3^Department of Surgical Sciences, University of Turin, Italy

^4^Ikazia Hospital, Department of Surgery, Montessoriweg 1, Rotterdam, The Netherlands

^5^Amsterdam UMC location University of Amsterdam, Department of Surgery, Meibergdreef 9, Amsterdam, The Netherlands.

^6^Erasmus MC, Department of Surgery, Dr. Molewaterplein 40, Rotterdam, the Netherlands.

**Corresponding author.** Alexander Grüter, address: Amsterdam UMC, location VUmc, De Boelelaan 1117, 1081 HV, Amsterdam.

**ORCID ID**: 0000-0002-6073-1003; **Twitter/X**: @alexandergruter

**Supplementary Materials - Index**

| **Supplementary Appendixes** |  |
| --- | --- |
| **Supplementary File 1.** Original study protocol entire RIGHT study | *See attached file* |
| **Supplementary File 2**. Original study protocol phase 1 RIGHT study | *See attached file* |
| **Supplementary Figures and Tables** |  |
| **Supplementary Table 1**. Patient characteristics | *pag. 2-3* |
| **Supplementary Table 2.** Postoperative complications | *pag. 4* |
| **Supplementary Figure 1.** Flowchart inclusions  **Supplementary Figure 2.** Specimens according to Benz | *See attached file*  *See attached file* |
|  |  |
|  |  |

**Supplementary Figures and Tables**

**Supplementary Table 1.** Patient characteristics

| Characteristic | Value |
| --- | --- |
| Gender  *Males*  *Females* | 198 (47.8%)  216 (52.2%) |
| Age (y) | 75 (IQR: 67-80, range: 38-94) |
| BMI (kg/m^2^) | 25.9 (IQR: 23.5-29.0, range: 15.5-46.3) |
| Medical history | 306/414 (73.9%) |
| Abdominal surgical history | 102/414 (24.6%) |
| Any previous malignancies | 60/414 (14.5%) |
| Anticoagulant therapy | 152/414 (36.7%) |
| Pre-operative defunctioning ostomy in situ  *No*  *Ileostomy*  *Colostomy* | 413 (99.8%)  1 (0.2%)  0 |
| ASA classification  *1: Fit & Healthy*  *2: Some illness, normal activity*  *3: Illness, some restriction* | 51 (12.4%)  202 (49.0%)  159 (38.6%) |
| pT stage  *pT1*  *pT2*  *pT3*  *pT4a*  *pT4b* | 32 (8.2%)  104 (26.8%)  202 (52.1%)  38 (9.8%)  12 (3.1%) |
| pN stage  *pN0*  *pN1a*  *pN1b*  *pN1c*  *pN2a*  *pN2b* | 277 (70.1%)  40 (10.2%)  33 (8.4%)  7 (1.8%)  21 (5.4%)  13 (3.3%) |
| Perforation | 3/379 (0.8%) |
| Type of tumour  *Adenocarcinoma*  *Mucinous carcinoma*  *Signet ring cell carcinoma*  *Medullary carcinoma*  *Undifferentiated carcinoma*  *Other* | 353 (89.9%)  22 (5.6%)  2 (0.5%)  6 (1.5%)  2 (0.5%)  8 (2.0%) |
| Differentiation grade  *Well/moderately differentiated*  *Poorly differentiated*  *Undifferentiated* | 296 (80.0%)  63 (17.0%)  11 (3.0%) |
| Lymphovascular invasion | 100/392 (25.5%) |
| Perineural invasion | 8/392 (2.0%) |
| Completeness of resection  R0  R1 | 391 (99.5%)  2 (0.5%) |
| Number of lymph nodes | 24 (IQR: 18-32, range: 0-99) |
| Microsatellite instability  *Normal: microsatellite stable (MSS)*  *Abnormal: microsatellite instability (MSI)*  *Not tested/unknown* | 83 (20.0%)  45 (10.9%)  286 (69.1%) |

*Values are presented as median (IQR and range) or number (%).*

**Supplementary Table 2.** Postoperative complications

| Complication | Value |
| --- | --- |
| Postoperative complications within 90 days  *Anastomotic leakage*  *Intra-abdominal abscess*  *Postoperative rebleed*  *Ileus*  *Wound infection*  *Fascial dehiscence*  *Bowel perforation*  *Ureter/bladder leakage*  *Negative re-laparoscopy*  *Negative re-laparotomy*  *Pulmonary complication*  *Cardiac complication*  *Thromboembolic complication*  *Infectious complication*  *Neurological complication* | 104/402 (25.9%)  11 (2.7%)  3 (0.7%)  14 (3.5%)  24 (6.0%)  10 (2.5%)  2 (0.5%)  2 (0.5%)  2 (0.5%)  7 (1.7%)  2 (0.5%)  19 (4.7%)  9 (2.2%)  5 (1.2%)  11 (2.7%)  5 (1.2%) |
| Overall Clavien-Dindo Classification  *Grade I*  *Grade II*  *Grade III*  *Grade IV*  *Grade V* | 29 (7.2%)  40 (10.0%)  27 (6.7%)  5 (1.2%)  3 (0.7%) |
| 90-day mortality | 3/402 (0.7%) |
| Time to tolerance of oral solid intake (days) | 1 (IQR: 0-1, range: 0-17) |
| Duration of hospital admission (days) | 3 (IQR: 3-5, range: 0-215) |
| Readmission | 42/401 (10.5%) |
| Intensive care admission | 14/402 (3.5%) |
| Nasogastric tube 5 days or more after the operation still in situ | 30/400 (7.5%) |
| Tube feeding started after the operation | 13/400 (3.3%) |

*Values are presented as median (IQR and range) or number*
