# Supplementary figures and images for: Nationwide variations in the execution of minimally invasive right hemicolectomy and short-term outcomes: first phase of the RIGHT study
Source: Br J Surg. 2024 Nov 18;111(11):znae291. doi: 10.1093/bjs/znae291 (PMC11572717; doi:10.1093/bjs/znae291)

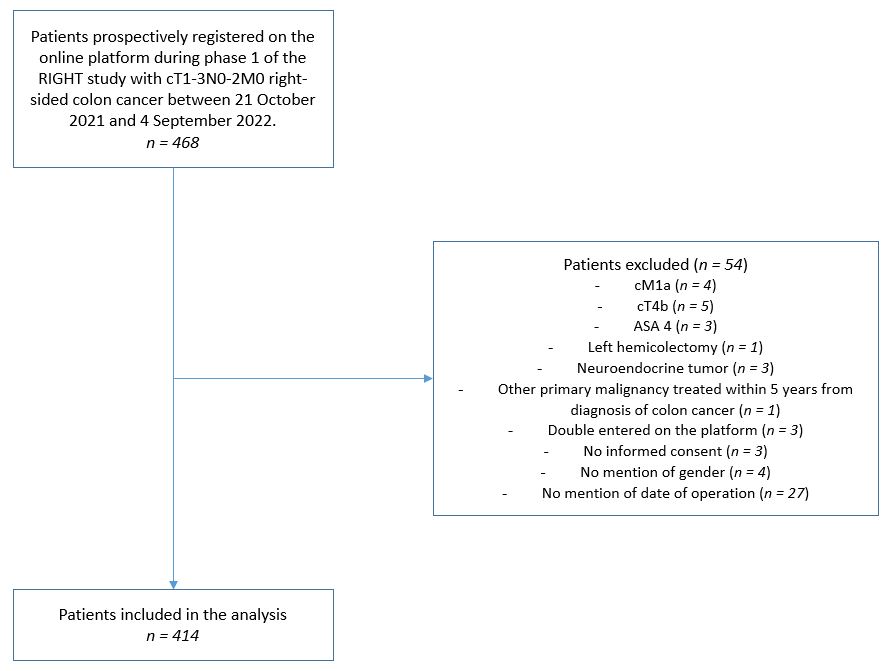

Supplement: znae291_Supplementary_Data [file znae291_supplementary_data.zip › Supplementary_Figure_1.JPG]

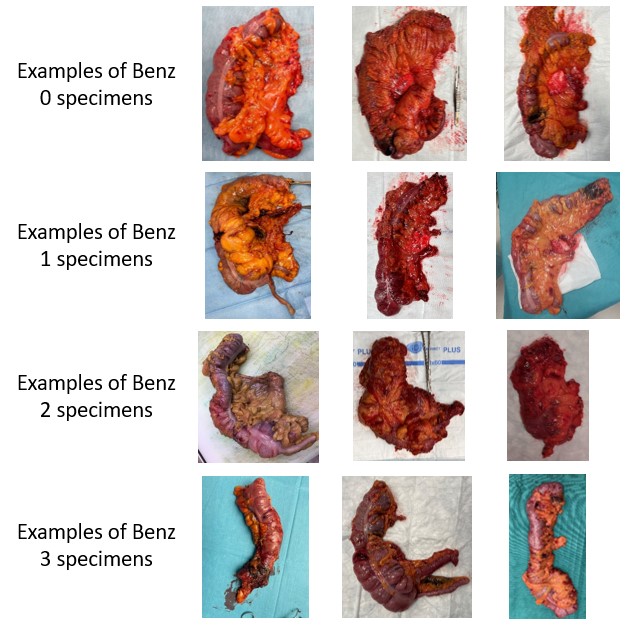

Supplement: znae291_Supplementary_Data [file znae291_supplementary_data.zip › Supplementary_Figure_2.jpg]
